# Nanoscale multi-beam lithography of photonic crystals with ultrafast laser

**DOI:** 10.1038/s41377-023-01178-3

**Published:** 2023-07-04

**Authors:** Jiaqun Li, Jianfeng Yan, Lan Jiang, Jiachen Yu, Heng Guo, Liangti Qu

**Affiliations:** 1 State Key Laboratory of Tribology in Advanced Equipment, Department of Mechanical Engineering, Tsinghua University, Beijing, 100084 China; 2 School of Mechanical Engineering, Beijing Institute of Technology, Beijing, 100081 China; 3 Department of Chemistry, Tsinghua University, Beijing, 100084 China

**Keywords:** Optical materials and structures, Lithography

## Abstract

Photonic crystals are utilized in many noteworthy applications like optical communications, light flow control, and quantum optics. Photonic crystal with nanoscale structure is important for the manipulation of light propagation in visible and near-infrared range. Herein, we propose a novel multi beam lithography method to fabricate photonic crystal with nanoscale structure without cracking. Using multi-beam ultrafast laser processing and etching, parallel channels with subwavelength gap are obtained in yttrium aluminum garnet crystal. Combining optical simulation based on Debye diffraction, we experimentally show the gap width of parallel channels can be controlled at nanoscale by changing phase holograms. With the superimposed phase hologram designing, functional structures of complicated channel arrays distribution can be created in crystal. Optical gratings of different periods are fabricated, which can diffract incident light in particular ways. This approach can efficiently manufacture nanostructures with controllable gap, and offer an alternative to the fabrication of complex photonic crystal for integrated photonics applications.

## Introduction

Manipulation of light propagation is an interesting field in optics and optoelectronics^1,2^. As one of basic components of integrated photonics, photonic crystals play an important part in the transmission and regulation of light^3–5^. Photonic crystals are a kind of structure consisting of a periodic array of uniform dielectric “atoms”. The uniform dielectric “atoms” can be air-filled holes, channels, dielectric rods, and other structures which have different refractive index with original materials. Due to the periodical arrangement of different refractive index structures, photonic crystals behave a considerable manipulation ability for light and photonics since its discovery^6,7^. Due to unique optical properties of photonic crystals, photonics devices can be constructed including light emitting devices, optical receivers, switchers, modulators, and resonators^8–10^. Photonic crystal structures in these optical devices are characterized as one-dimensional (1D), two-dimensional (2D), and three-dimensional (3D) according to lattice arrangements. Different from the 1D and 2D cases, the fabrication of 3D structure is rather difficult because the refractive index should be varied at three spatial directions^11,12^.

Laser lithography is one of potential techniques for 3D structure fabrication in crystals. It is known that ultrafast laser is a versatile tool used for marking, cutting, patterning, and structuring. Due to the ultrahigh intensity, ultrafast laser can overcome bandgap of crystals and realize direct micromachining^13^. The ultrashort pulse duration also makes ultrafast laser lithography a non-thermal processing method, which can be used in many applications like aerospace, energy^14^, and optoelectronics^15^. Recently, femtosecond laser is employed to fabricate photonic crystals because of its processing ability for micro-nanostructures^16,17^, especially three-dimensional fabrication in transparent materials^18^. Femtosecond laser can induce physical and chemical changes in focus region due to well confinement of energy, which originated from the ultrafast properties^19–21^. Ultralow-loss geometric phase optical elements like prism, lens, and vector beam convertors can be fabricated through a new type of laser modification inside silica glass^22^. After laser processing, post-treatment like chemical etching^23^ and annealing^24^ can help to form hollow structures in laser-affected areas. Based on laser-induced structural changes, many photonic crystal structures can be fabricated in transparent dielectrics such as glass, sapphire, and Lithium niobite^25–27^. A micro-structured optical waveguides was fabricated in a single-crystal by femtosecond laser inscribing and acid etching, which can realize midinfrared waveguiding with a low loss^28^.

The size and spacing of structure in photonic crystals can greatly affect the performance of light manipulation^29^. To realize the control of light propagation, it is required that the structure of photonic crystal is comparable to its working wavelength. For photonic crystals with a bandgap at microwave range, the characteristic size needs to be controlled at centimeters scale. A negative refraction phenomenon of microwaves can be found in a metallic photonic crystal, which is consisted with centimeters rods^30^. For Terahertz (THz) wave and midinfrared wave range, photonic crystal structure should be micrometers. An integrated platform was fabricated based on photonic crystal waveguides of micrometers lattice constant, which can be used in terahertz communications^31^. Studies have shown the fabrication of photonic crystal with nanoscale structure is important to realize visible and NIR light manipulation^32,33^.

In femtosecond laser processing, the realization of nanoscale structure was mainly restricted by optical diffraction limit. Recently, some works have reported the fabrication of nanostructures at tens of nanometers by laser direct writing lithography^34^. Nanogrooves with a minimum feature size down to 30 nm can be formed directly on silicon surface with pulsed nanosecond laser irradiation^35^. Straight large-area nanoscale laser-induced periodic surface structures was fabricated with two femtosecond laser beams^36^. These methods are still limited to planar and non-transparent materials. The structure can only be fabricated on the sample surface. It is necessary to propose a more suitable method for the processing of three-dimensional nanostructure inside crystals. To process three-dimensional structures in transparent dielectrics, it is known that femtosecond laser-induced structure can break diffraction limit by tight focusing, multiphoton effect, and other nonlinear processes^37,38^. These methods use conventional single-beam direct writing and require multiple scanning. Thus, the laser spot overlapping and movement accuracy are imperative to be considered in femtosecond laser direct writing. In conventional single-beam processing, multiple scanning method is employed to fabricate complicated structures. The laser-affected region overlapping will induce secondary processing, which limits the reduction of structure size. Besides, the accuracy and stability of mechanical movement will influence fabricated structures. Multiple scanning requires accurate control over relative motion between laser and crystals.

To improve the capacity of conventional single-beam processing method, parallel laser processing techniques, such as microlens array fabrication (MLA) and laser interference lithography (LIL), have been developed. MLA lithography enables the rapid fabrication of large-scale micro/nano pattern arrays on substrate surfaces^39,40^. However, a single micro lens array can only produce a specific multi-focus array. Micro lens arrays with varying morphologies and distributions must be fabricated for different micro-nanostructures processing demand. Besides, it is required to design each micro lens if the focus of multi-beam field have a special distribution. LIL technique generates interference patterns of two incident laser beams and prints patterns onto materials to directly form micro/nanostructure arrays^41,42^. While LIL offers the advantage of easily switching micro/nanomachining patterns, it is generally limited to the fabrication of planar structures. Therefore, a processing method with 3D manufacturing capability and easy adjustment is necessary. In this work, a multi-beam laser lithography strategy based on beam shaping is proposed to realize customizable three-dimensional nanostructure fabrication in crystals.

Here, we show parallel channels with nanoscale gaps can be realized in yttrium aluminum garnet (YAG) crystal by tightly focusing of multi-beam femtosecond laser. The multi-beam laser direct writing process is performed by spatial light shaping with an optical phase hologram applied on a spatial light modulator (SLM). Laser-modified region is etched to obtain hollow channel structures after direct writing. The distance between parallel channels can be varied from hundreds of nanometers to several micros by changing the SLM phase holograms distribution. Meanwhile, the laser fabrication efficiency is also promoted due to the multi-beam parallel processing, which is demonstrated through 4-channels and 8-channels fabrication. Complicated channel arrays can be produced with multi-beams distribution control. This is achieved by superimposed phase hologram design. Based on the multi-beam lithography method, optical devices like diffraction gratings are created in crystals. The diffraction test of fabricated gratings is consistent with theoretically analyzed results. This method allows precise and efficient fabrication for nanoscale structure in photonic crystals.

## Results

### Multi-beam laser direct writing method

**Fig. 1:**
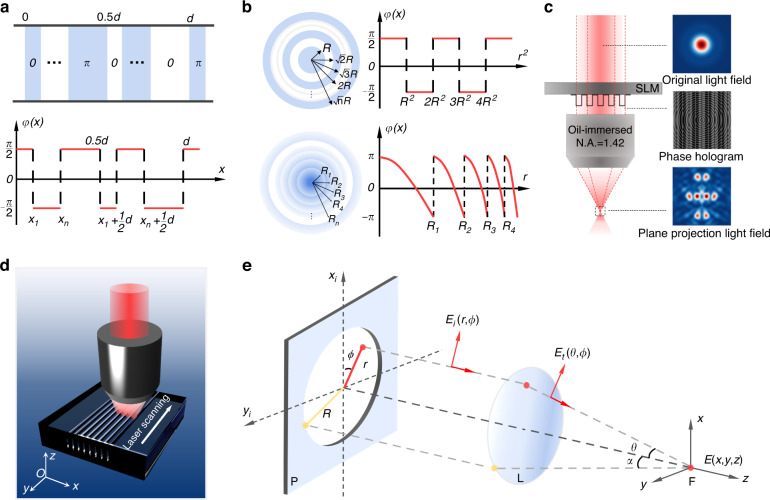
**Schematic illustration of multi-beam processing in YAG crystal with SLM-based femtosecond laser system.** **a** SLM holograms used to generate a simple multi-beam light field. **b** Description of hologram phase for complicated light field. **c** Multi-beam light field shaping with superimposed phase hologram. **d** Diagram of multi-beam scanning strategy. **e** Diagram of Debye diffraction integral, *P* represents the pupil, *L* represents the objective lens, and *F* represents the focusing region

The principle and experiment detail of multi-beam laser direct writing was summarized and displayed in Fig. 1. As shown in Fig. 1a, a Dammann grating phase was used to generate simple multi-beam light field. Adjusting the turning point of binary phase, the spacing and numbers of multi-beam focus can be controlled. Figure 1b presents the original and optimized Fresnel phase used to reorganize the multi-beam focus spatial distribution. Combining Dammann grating phase and Fresnel phase, we apply the superimposed phase hologram onto SLM as Fig. 1c describes. A complicated multi-beam light field consisted of 8-focus spots in x-z plane can be obtained. As Fig. 1d displays, a multi-channel array can be modified by using multi-beam scanning method in a transparent crystal sample. After laser scanning, the modification areas were etched by acid from hollow channels which is detailed described in Method. To theoretically analyze the multi-beam light field, Debye diffraction integration was employed to calculate optical intensity distribution in a tightly focused condition as Fig. 1e shows.

### Parallel channels fabrication with controllable nanoscale gap

**Fig. 2:**
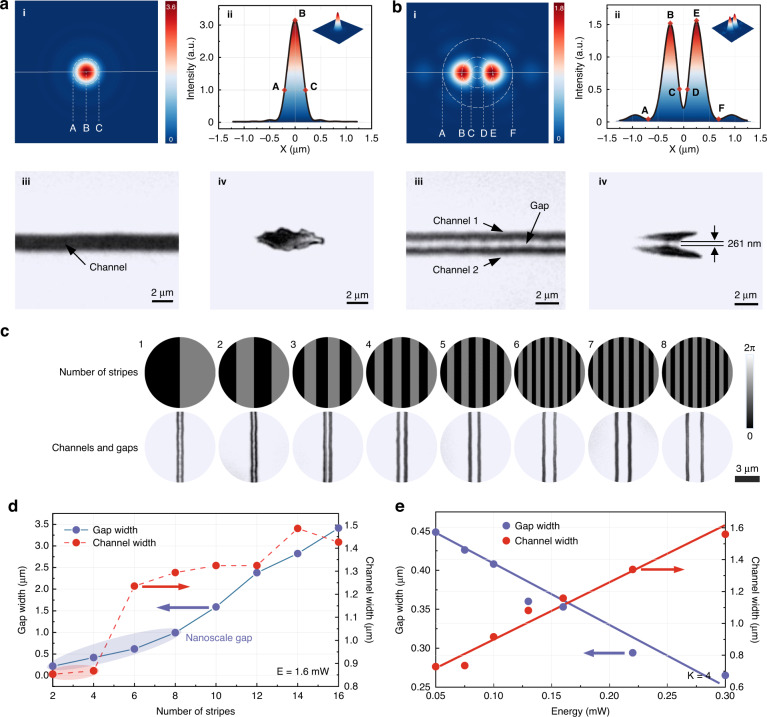
**Demonstration of double-channel processing.** **a** i) Simulated intensity distribution of single beam in x–y plane; ii) Intensity profile of single beam; iii) top view optical micrography of conventional single beam processing result; iv side view optical micrography of conventional single beam processing result. **b** i) Simulated intensity distribution of double beams in x–y plane; ii Intensity profile of double beams; iii) top view optical micrography of double beams processing result; iv) side view optical micrography of double beams processing result. **c** SLM phase holograms and experimental results for different stripe numbers *K*. **d** Measured gap and channel width against different stripe numbers *K*. **e** Measured gap and channel width against different pulse energy

The crystal is modified with conventional single beam scanning method and our proposed multi-beam scanning strategy. The laser energy of each beam is controlled higher than modification threshold of YAG crystal but lower than its ablation threshold. Thus, the laser scanning process only induce structural change rather than material removal or ripple formation. After laser inscribing, all the processed area was etched with acid to form channels. Our experiments utilized the transverse writing scheme which can abandon the constraints of the lens working distance, despite asymmetric focus spot. Due to the non-circle laser focus spot, the cross-section of channels is not a standard circle. Figure 2 displays the etching result after traditional single beam and proposed multi-beam laser irradiation. A tightly focused light field was utilized to form modified line tracks with a 20 μm s^−1^ scanning speed. Figure 2a shows the light field simulation and etched single channel after unshaped single beam laser processing. A single straight channel with about 1μm width can be seen. The measured channel width is a little larger than the simulated diameter *X*_*AC*_. Figure 2b presents the etched double channels processed with a two-beam light field. Channel 1 and Chanel 2 can be seen from top view of optical microscope, and quite distinct with each other. The width of channel was measured ~0.7 μm. A narrow gap down to 261 nm width can be seen clearly both in top view and side view image. The simulation provides an estimate of gap width *X*_*CD*_, which is about 200 nm. The calculation is almost equal to the experiment result. The difference between simulated and experimentally measured results is mainly due to that the energy at CD points is lower than the modification threshold of crystal, resulting in the width of the actual etched area is smaller than the simulated value. It can be found that there is no apparent difference between two channels and their surrounding areas. Thus, we can infer the multi-beam parallel scanning strategy help to get uniform subwavelength photonic crystals structures without secondary processing or mechanical fluctuations which displayed in Fig. S3.

Applying optical holograms on spatial light modulator allows to fabricate multi channels with a high freedom. By changing the stripe numbers of binary phase mask hologram, the distance between two-beam focus spots can be adjusted. In Fig. 2c, we can see the gap width has a positive correlation with stripe numbers of grating phases. When the stripe numbers increase from 2 to 16, the distance between two channel increases gradually. Figure 2d shows a more detailed analysis for the gap width and channel size. When the stripe numbers *K* are <8, the size of gap remains at nanoscale. With stripe numbers increasing, gap size behaves a linearly increasing relationship. Meanwhile, it can be observed that only *K* = 2 and *K* = 4 can obtain a channel with width less than 1 μm. When the stripe numbers are 6, the channel width has an abrupt promotion. The pulse energy during laser writing can also influence the gap and channel width. Figure 2e gives the result of processed nanostructures with pulse energy varied from 50 to 300 nJ. The gap width has a negative relationship with pulse energy when the channel width behaves positive.

For larger size up to several micros, the relationship between gap width and stripe numbers was summarized in Fig. S4. There is an interesting phenomenon that the gap width increases and the cross-section length decreases when the number of interference stripes increases. It is believed that the phenomenon can be explained by Fraunhofer diffraction theory and can be predicted with Debye diffraction integral simulation. When the stripe numbers increase from 18 to 72, the distance between adjacent channels increases from 6 to 18 μm. The experiment results taken from top view and side view are well consistent with optical simulation. Changing the phase value of hologram on SLM can adjust the relative size of two focus as Fig. S5 displayed. Based on channel size and gap width control, a costumed binary mask hologram can be designed, allowing the fabrication of microchannel arrays with arbitrary size and gap width.

To expand the efficiency and flexibility of multi-beam direct writing method, other Dammann grating phase holograms are designed to generate desired light field distribution for more channels. Fig. S6 shows 2,4,8-beam light field simulation and processed results, respectively. Designed Dammann grating phase holograms and corresponding light field simulation were presented. Applying the grating phase, two, four, and eight parallel tracks can be processed with multi-beam direct writing. We show the laser-modified tracks behave different refractive index compared to untreated area, which can be seen as a darker region. After phosphoric acid treating, the dark region was etched to form hollow channels. From side and top view of optical image, the cross-section of etched channels both shows a uniform distribution. By using the N-beam light field, the efficiency of laser scanning process was raised by N-1 times.

## Discussion

### Structure analysis of modified crystals

**Fig. 3:**
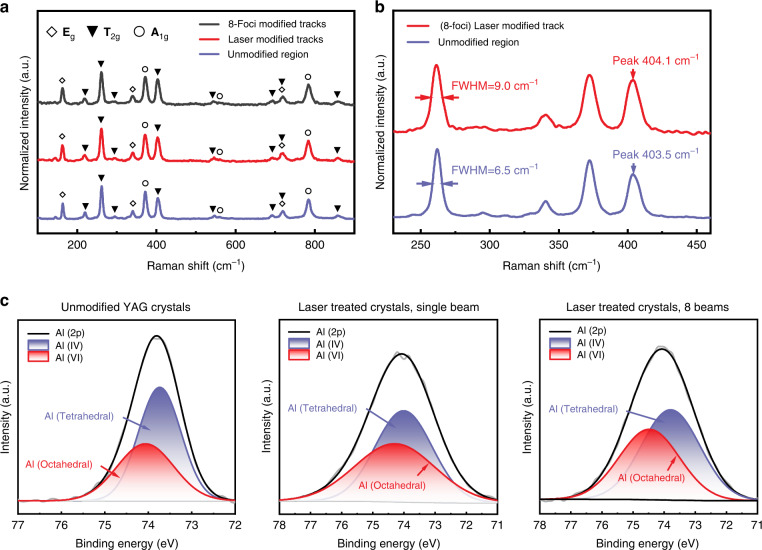
**Structure characterization of multi-beam direct writing results.** **a** Raman intensity spectrums of laser processed area and unmodified region. **b** Enlargement of Raman intensity spectrums for wavenumbers from 230 cm^−1^ to 460 cm^−1^. **c** XPS intensity of multi-beam processed area and unmodified region

In order to further validate the capabilities of proposed method, structure changes of laser-modified areas are studied. Figure 3a displays Raman spectrum of unmodified region and laser-modified tracks in crystals. There is no apparent difference between laser-modified tracks (blue and red lines) and unmodified region (black lines) in most area. An intensity increment of the 403 cm^−1^ T_2g_ Raman mode can be found, which represents the increase of aluminum ion in octahedral site. In Fig. 3b, the Raman mode of laser-modified tracks behaves a slight blue shift (i.e., from 403.5 cm^−1^ to 404.1 cm^−1^) and FWHM broaden (i.e., from 6.5 cm^−1^ to 9 cm^−1^ at 263 cm^−1^). The two changes of Raman spectrum may imply the presence of lattice defects such as partial damage and bond broken in laser-processed crystal due to crystal transformation as previous research has reported in similar crystals^43^. The higher chemical reactivity caused by defects is the reason for the chemical etching rate difference between laser-modified tracks and untreated region^44,45^. It can also be seen that the Raman spectrum of single-beam and 8-beam processed area tracks are almost the same.

X-ray photoelectron spectroscopy (XPS) was used to characterize the chemical state of crystal components. XPS intensity of Al_2p_ spectrum in 8-beam processed region and unmodified region are shown in Fig. 3c. The Al ion spectrum of unmodified crystal can be divided into two contributions, the octahedral site (Al_VI_) and tetrahedral site (Al_IV_), located at respectively 74.27 eV and 73.78 eV. The relative ratio of peak area is 2:3, consistent with the unit cell of garnet structure. After laser inscribing, the peak area of Al_VI_ was found to have an increase when the peak area of Al_IV_ suffered a decline. It can be attributed to the transformation of Al ions from tetrahedral site to octahedral site, which is similar in the Raman spectrum results. The XPS intensity of 8-beam laser-modified region is almost the same as traditional single-beam written results. It means multi-beam processed regions have no difference in structures or components with single beam fabricated one. Based on Raman and XPS analysis, the uniformity (the interval of processed channels remains invariable) and stability of multi-beam direct writing method can be seen, which means the approach can offer possibilities for efficient fabrication of channel arrays with a determinant size.

### Complicated structure fabrication using superimposed SLM phase

**Fig. 4:**
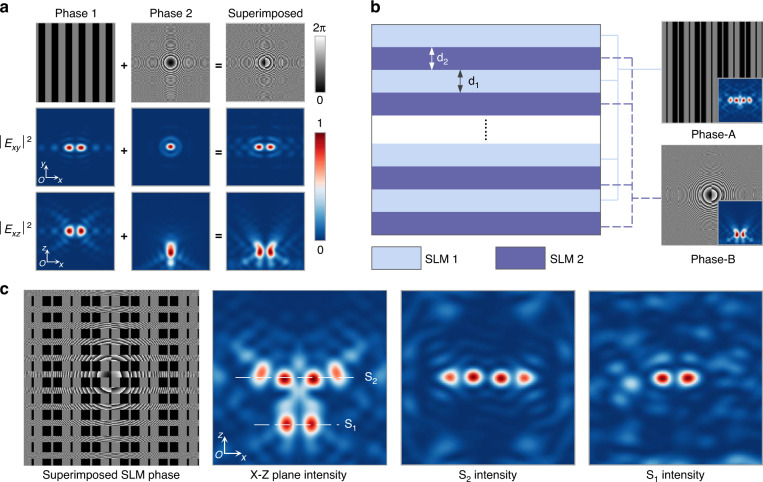
**Schematic illustration of superimposed SLM holograms for complicated multi-beam generation.** **a** Phase holograms and simulated intensity for Dammann grating phase, Fresnel phase, and superimposed phase. **b** Diagram of SLM-split-use approach and phase holograms applied to sub-SLMs. **c** Final composed SLM phase hologram and simulated optical intensity

Aiming to generate the more complicated light field, a superimposed SLM hologram was used. As Fig. 4a shows, a simple binary Dammann grating phase can be applied to get two-beam focus spots split along x-y plane. An optimized blazed Fresnel phase can be calculated to generate a spot away from the original focus plane along the propagating direction. When the two phase was combined, two focus spots deviated from the original position can be obtained in x-z plane. In Fig. 4b, we proposed a SLM-split-use approach to generate aimed laser intensity distribution. The SLM was divided into several strip areas which can be used as sub-SLMs to apply different phase holograms. The strip width d_1_ and d_2_ can be adjusted to balance the multi-beam focus intensity.

In this experiment, Phase-A applied to SLM1 is a Dammann grating phase that can generate four-beam focus spots at focusing plane of objective lens. Phase-B applied to SLM2 is a superimposed phase combining a Dammann grating phase and a blazed Fresnel phase that can generate two spots away from the focal plane. The simulated optical intensity distribution of x-z plane for each phase was shown in bottom right insert image. The ratio between d_2_ and d_1_ was set as 3:4. The final superimposed SLM hologram was shown in Fig. 4c. Debye diffraction integration was done to simulate the optical intensity of x-z plane. A combination of 4 beams in the central part and 2 beams offset the focus plane can be seen. The intensity of S1 and S2 at x-y plane were also calculated. Although each sub-SLM only diffracts part of the incident laser, the focus result of each phase hologram was found barely affected. For more channel array fabrication, the number of sub-SLMs should be expanded to produce desired plane intensity distribution. It is worth noting that other SLM-split-use approach can also be used to generate superimposed light field as Fig. S8 presents. In this case, each angle of sub-SLMs need to be designed specially.

**Fig. 5:**
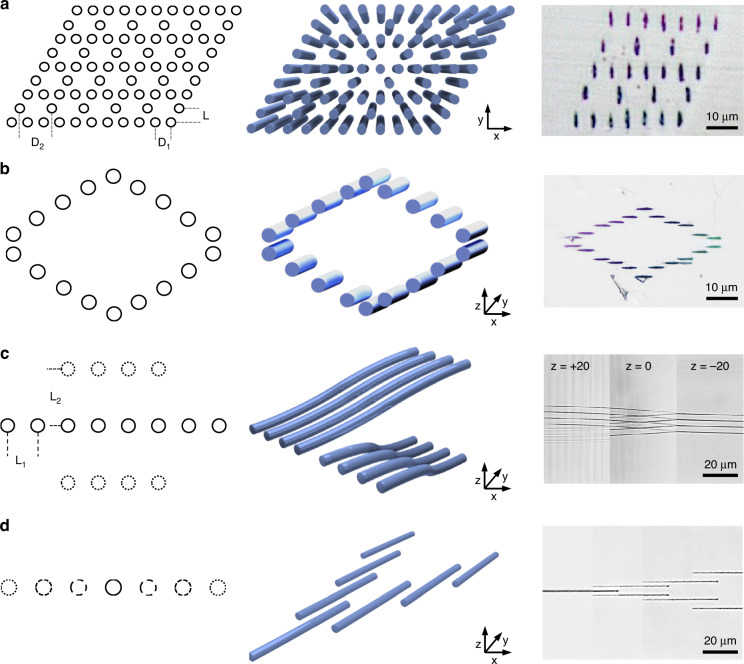
**Design and fabrication of different complicated structures in crystals.** **a** Plane and 3D schematics, optical microscope image for the parallelogram-distributed structures. **b** Plane and 3D schematics, optical microscope image for the rhombus-like structures. **c** Plane and 3D schematics, optical microscope image for a 2 × 4 beam splitter. **d** Plane and 3D schematics, optical microscope image for a light divider element

As shown in Fig. 5, multi-channel arrays with different spatial distribution are processed. A parallelogram-distributed channels array is displayed in Fig. 5a. The surface morphology is experimentally captured. The incident light can be coupled to the fabricated structure from a corner (bottom left) and the output from another corner (top right) can be observed if the parameters are specially designed. Figure 5b shows a rhombus-like channel array with a central unmodified area. The central area is surrounded by processed tracks and can serve as a cladding waveguide. Other waveguiding structures can also be fabricated with the multi-beam direct writing method. A 2 × 4 spatial beam splitter structure is presented in Fig. 5c. The incident light passing through the binding waveguide structure can be divided into a 2 × 4 light arrays of almost the same intensity. In Fig. 5d, a bulk light divider based on evanescent coupling can be prepared by multi-beam scanning with varied channel spacing. When the length of each channel satisfies the evanescent wave coupling condition, the incident light from a single input can be divided into two output beams (~1:1 energy ratio).

### Fabrication of diffraction gratings

**Fig. 6:**
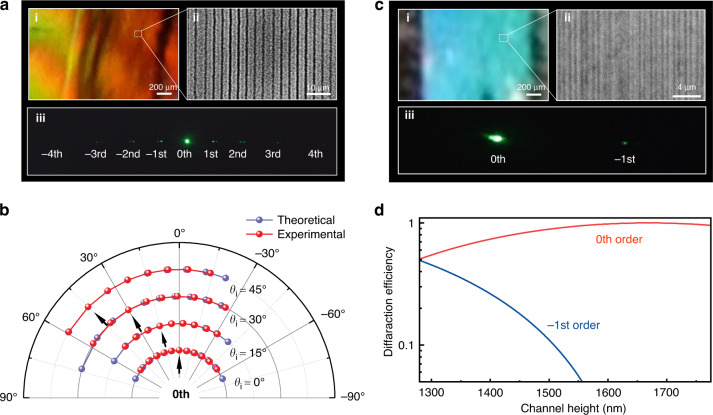
**Demonstration and characterizations of processed optical gratings.** **a** Gratings with a period of 3.5 μm. i) Grating image under white light illumination. ii) Optical microscope image of fabricated long-period gratings structures. iii) Diffraction pattern with green light illumination. **b** Analysis and test of fabricated gratings with illumination from different incident angles. **c** Grating structure with a subwavelength period of about 600 nm. i) Grating image under white light illumination. ii) Optical microscope image of fabricated subwavelength gratings structures. iii) Diffraction pattern with green light illumination. **d** Theoretical analysis of diffraction efficiency

The multi-beam processing approach provides potentials of fabrication for a variety of photonic crystals elements. Figure 6a shows a diffraction grating processed by 8-beam light field. A multicolor distribution on the fabricated crystal can be observed under a white light illumination. The fabricated grating consists of channels with 3.5 μm gap spacing as can be seen in insert image ii. Theoretically, the grating structure allows to diffract incident green light into 13 diffraction orders from -6th to 6th. Due to the weak intensity of higher diffraction orders, only -4th to 4th orders can be photographed in image iii. Varying the incident angle of illumination light, the diffraction angle of each diffraction order changes, which is recorded by a CCD camera in Fig. 6b. According to the diffraction equation, the diffraction angle can be calculated almost coincide with the experimental results.

Subwavelength grating can induce light response in a particular way which is different from traditional gratings. For a visible or near-infrared light luminescence source, the characteristic size of subwavelength grating needs to be hundreds of nanometers. By applying multi-beam light field with nanoscale beams interval, it can be seen a subwavelength grating structure is obtained as Fig. 6c displayed. The insert image iii shows only 0th and -1st order diffraction green light can be observed after passing through the grating structure. The relative intensity of 0th and -1st order light is mainly dependent on the size of grating structure channel elements and incident wavelength. Simulation of channel size influence based on *n*_neff_ was presented in Fig. 6d.

Other optical elements like waveguide and beam splitter can also be fabricated using the multi-beam parallel processing method. A photonic crystals micro-structured optical waveguide was shown in Fig. S10a schematically. Hexagon distribution of hollow channels with size of 1.2 × 0.6 μm^2^ can be seen and the central area with a diameter of 3 μm can be used to guide incident light. Fig. S10b shows the simulation of intensity mode profile for the waveguide under 800 nm light luminescence. The distribution of the mode electric field component and power flow density with 800 nm light luminescence was calculated in Fig. S10c.

In summary, a multi-beam direct writing method followed by etching is reported to realize nanoscale structure lithography in crystals. The structure consisted of multi-channel arrays with subwavelength gaps. The laser direct writing process was performed by spatial light shaping with a SLM phase hologram. Dammann grating phase was used to generate multi-beam light field which can modify the area to be etched. Parallel channels with subwavelength gap of 261 nm are created in YAG crystal. Gap width can be varied by changing the interval between multi-beams through phase distribution control. To process complicated channel arrays, Blazed Fresnel phase was utilized to reorganize the spatial distribution of multi-beam focus. Combining the SLM-spilt use approach, superimposed phase hologram was designed to fabricated complicated channel array structure with multi-layers. Based on the method, optical gratings of nanoscale gaps were processed which can diffract incident light in a particular way. The flexible control of nanogap between parallel channels makes the approach an alternative to weave complex photonic crystals with subwavelength structure. The potentials of multi-beam processing method may open possible ways to fabricate nanostructure for applications in optical communication and light manipulation.

## Materials and methods

### Phase holograms design

The multi-beam light field was generated by applying holograms on a 1920 × 1080 pixels liquid-crystal spatial light modulator (LC-SLM). To obtain a simple multi-beam laser beam, a binary Dammann grating phase was applied to the liquid-crystal screen of SLM. The grating phase consists of two kinds of phase values (0 and *π*). The variation of the multi-beam light field is achieved by changing the position of the phase value turning point. The position and the number of binary phase pattern dividing lines are designed specifically to realize control of multi-beam distribution. On one-dimensional condition, when a single period of an even type Dammann grating is irradiated by monochromatic light, the relative amplitude of each diffraction order can be described by

$$\begin{array}{l}A(n)=\frac{1-{(-1)}^{n}}{\pi n}\mathop{\sum }\limits_{k=1}^{N/2}{(-1)}^{k}\exp (-2\pi n{x}_{k}) \\\qquad\quad\;\;\varphi ({x}_{k}+d/2)=\varphi ({x}_{k})+\pi\end{array}$$

where *n* is the diffraction order, *d* is the period of Dammann grating, *N* is the number of phase pattern dividing lines in the first half period.

In above equations, ${x}_{k}$ is the coordinate of the phase step, which can be solved numerically by simulated annealing algorithm or global gradient method. In the second half period, the phase step coordinates can be described by adding a *π* phase to the first half, as shown in equation (2). When a two-beam light field distribution is needed, the number of dividing lines *N* is 2. Calculated phase step coordinate *x*_1_ is 0.5 if we set the period d as 1. When a four-beam light field distribution is needed, the number of dividing lines *N* should be 4. The phase step coordinates *x*_1_, *x*_2_ are calculated as 0.22057 and 0.44563.

To realize more complicated structure lithography in crystals, a light intensity distribution at x-z plane or y-z plane is required. It is known that the position of light focus will have an offset along the propagation direction with a Fresnel phase. Fresnel phase is consisted with a series of concentric circles. The function of Fresnel phase can be written as

$${\varphi }_{{\rm{Fresnel}}}=\frac{\pi }{2}+\frac{\pi }{2}\cdot {\rm{signum}}\left(\frac{r}{8\times {10}^{-6}}\,\mathrm{mod}\,40-20\right)$$

where r is the location variable in polar coordinate.

When a Fresnel phase was superimposed to the original Dammann grating phase, a planar light intensity distribution in x-z plane was obtained. It can be found that two focus spots split along the propagating direction. By optimizing the Fresnel phase distribution, the beam focus spot can only move away or approach along a single direction. The function of optimized blazed Fresnel phase used in the experiment is

$${\varphi }_{{\rm{Blazed}}{\rm{Fresnel}}}={\left[\frac{2\pi }{\lambda }(\sqrt{{f}^{2}+{r}^{2}}-f)\right]}_{2\pi }$$

### Experimental setup

A SLM-based laser direct writing system is used in experiment. The ultrafast laser source was an amplified Ti: sapphire femtosecond laser system, with a central wavelength of 800 nm, pulse duration of 35 fs and repetition rate of 1 kHz. A liquid-crystal spatial light modulator was used to shape the laser intensity distribution by applying phase holograms. A 4 f system consisted of two convex lenses was utilized to eliminate the diffraction effect before laser focusing. The focus length of two convex lens is 400 mm. The shaped laser was tightly focused with an oil-immersed microscope objective. The numerical aperture (NA) of objective lens was 1.42 and the working distance was 0.15 mm.

### Debye diffraction integration

The light field distribution was simulated with vectorial Debye diffraction theory for tightly focused condition. According to the Richards-Wolf vectorial diffraction integration method, the electric field intensity of a certain location in focusing region can be described as

$$\begin{array}{l}E(x,y,z)=A{\int }_{0}^{\alpha }{\int }_{0}^{2\pi }P(\theta ){E}_{t}(\theta ,\phi )\exp (ikz\,\cos \theta )\sin \theta \times \\\qquad\qquad\quad\; \exp \{-ik\sqrt{{x}^{2}+{y}^{2}}\,\sin \theta \,\cos \theta [\arctan (y/x)-\phi ]\}d\phi d\theta \end{array}$$

*A* is a constant determined by focus length and wavelength, *P(θ)* is apodization function, and wavenumber *k* is defined as *2π/λ*. *θ* is refraction angle of objective obtained by formula *sinθ* = *rNA/(Rn*_*t*_*)*, where *R* is beam radius at the incident pupil, *NA* is the numerical aperture of objective, and *n*_*t*_ is the refractive index of focusing medium. *r* and *ϕ* is the polar coordinates at the incident plane. *x, y, z* is the cartesian coordinates in the focusing region.

*E*_*t*_ *(θ, ϕ)* describes the transmitted field of incident light passing though the objective lens, which can be calculated with the incident light field intensity *E*_*i*_ *(r, ϕ)*. In the actual calculation, the transmitted intensity *E*_*t*_ *(θ, ϕ)* can be described by

$$\left[\begin{array}{c}{E}_{tx}\\ {E}_{ty}\\ {E}_{tz}\end{array}\right]=\sqrt{\cos \theta }\left[\begin{array}{ccc}1+(\cos \theta -1){\cos }^{2}\phi & (\cos \theta -1)\cos \phi \,\sin \phi & -\,\sin \theta \,\cos \phi \\ (\cos \theta -1)\cos \phi \,\sin \phi & 1+(\cos \theta -1){\sin }^{2}\phi & -\,\sin \theta \,\sin \phi \\ \sin \theta \,\cos \phi & \sin \theta \,\sin \phi & \cos \theta \end{array}\right]\left[\begin{array}{c}{E}_{ix}\\ {E}_{iy}\\ {E}_{iz}\end{array}\right]$$

In above equation, *E*_*tx*_*, E*_*ty*_*, E*_*tz*_ are three orthogonal components of transmitted light passing through the objective lens in a cartesian coordinate system, when *E*_*ix*_*, E*_*iy*_*, E*_*iz*_ are three orthogonal components of the incident light.

Considering the focusing system satisfied Abbe’s sine condition, the final electric field intensity can be described as a function of wave vector ***k***.

$$E(x,y,z)={\int }_{0}^{\alpha }{\int }_{0}^{2\pi }P(\theta ){E}_{t}(\theta ,\phi )\exp [i({k}_{z}z-{k}_{x}x-{k}_{y}y)]\,\sin \theta d\phi d\theta$$

The wave vector ***k*** is defined as *k*_*x*_*e*_*x*_*+k*_*y*_*e*_*y*_*+k*_*z*_*e*_*z*_, where *k*_*x*_ = *−kcosϕsinθ*, *k*_*y*_ = *−ksinϕsinθ*, *k*_*z*_ *=* *kcosθ*. If we let *ξ* = *−cosϕsinθ/λ* and *η* = *−sinϕsinθ/λ*, the above equation can be written as

$$E(x,y,z)=\iint P(\theta ){E}_{t}(\theta ,\phi )/\,\cos \theta \times \exp (i{k}_{z}z)\exp [-i2\pi (\xi x+\eta y)]d\xi d\eta$$

which means the electric field intensity in the focusing region is Fourier transformation of the transmitted field intensity passing through the objective lens.

In this paper, light field simulation is based on above two equations. The phase and polarization information are coupled into the incident laser distribution with a vector expression of light field. Three orthogonal components *E*_*x*_, *E*_*y*_, *E*_*z*_ at the focusing region can be obtained, separately. The final optical intensity is the sum of the square of three components.

### Laser lithography

A 10 × 5 × 0.5 mm^3^ YAG crystal with crystal orientation <111> was used in the experiment. The focused laser spot was controlled about 100 μm beneath the surface. The refractive index of YAG crystal is 1.82 and the refractive index of silicone oil we used is 1.42. Considering the refractive index difference between YAG crystal and silicone oil, the depth of laser focus spot can be calculated as 100 × 1.82/1.42 = 128 μm. In the experiment, the line scanning process was performed in a plane perpendicular to the laser propagation direction. Fig. S1 displays the relationship between processed tracks and pulse density, which is determined by line scanning speed. To get a consecutive and uniform modified area, the scanning speeds of laser were set as 20 μm s^−1^.

The laser scanning process is performed in liquid environment. The interface between oil and crystal may cause fluctuations of laser light field and discontinuity of modified trajectory. Further polishing with alumina suspension was performed to expose the inner processed structures before chemical etching. Wet etching was implemented with hot phosphoric acid (H_3_PO_4_, 44%wt solution) in deionized water. The processed sample was etched with a magnetic stirrer heated at 75 °C for hours. The residual acid was removed by ultrasonic baths in deionized water and ethanol, respectively. The etching result after 80 h and 300 h was shown in Fig. S2. From the top view of the optical microscope image, an average etching rate of 21.25 μm h^−1^ can be calculated.

### Characterization

Optical microscope images of crystal samples were captured under a bright-field Olympus optical microscope. For the Raman spectra measurement of samples, a continuous laser (532 nm) was used as an excitation source. Raman shift varies from 100 cm^−1^ to 900 cm^−1^. X-ray photoelectron spectroscopy was utilized to obtain the change in crystal structures.

## Supplementary information

Supplementary Information for Nanoscale Multi Beam Lithography of Photonic Crystals with Ultrafast Laser
