# Supplementary material for: Nanoscale multi-beam lithography of photonic crystals with ultrafast laser
Source: Light Sci Appl. 2023 Jul 4;12:164. doi: 10.1038/s41377-023-01178-3 (PMC10317996; doi:10.1038/s41377-023-01178-3)
Supplement: Supplementary file 1 — Supplementary Information for Nanoscale Multi Beam Lithography of Photonic Crystals with Ultrafast Laser [file 41377_2023_1178_MOESM1_ESM.docx]

**Supplementary Information for**

**Nanoscale Multi Beam Lithography of Photonic Crystals with Ultrafast Laser**

*Jiaqun Li**^1,†^, Jianfeng Yan^1,†^, Lan Jiang^2,*^, Jiachen Yu^1^, Heng Guo^1^, Liangti Qu^3^*

^1^State Key Laboratory of Tribology in Advanced Equipment, Department of Mechanical Engineering, Tsinghua University, Beijing 100084, China

^2^School of Mechanical Engineering, Beijing Institute of Technology, Beijing 100081, China

^3^Department of Chemistry, Tsinghua University, Beijing 100084, China

**Corresponding authors:* [*jianglan@bit.edu.cn*](mailto:jianglan@bit.edu.cn)

*† These authors contributed equally to this work.*

1. **Laser direct writing result by single beam**

The pulse density of laser direct writing is determined by laser scanning speed. The optical microscope image of laser modified areas by different pulse energy are displayed in **Fig. S1a**. The scanning track is discontinuous when the pulse density is smaller than 10 pulse·μm^-1^. It can also be seen in **Fig. S1b** that the track is much wider when the pulse density is larger. Besides, there are many irregular structures originated from Gaussian distribution of pulse energy around the laser scanning tracks. The larger modified area and irregular structure around mean that it is more difficult to control the width of laser scanning result so that it does harm to the fabrication of nanostructure with nano gaps in crystals.

**
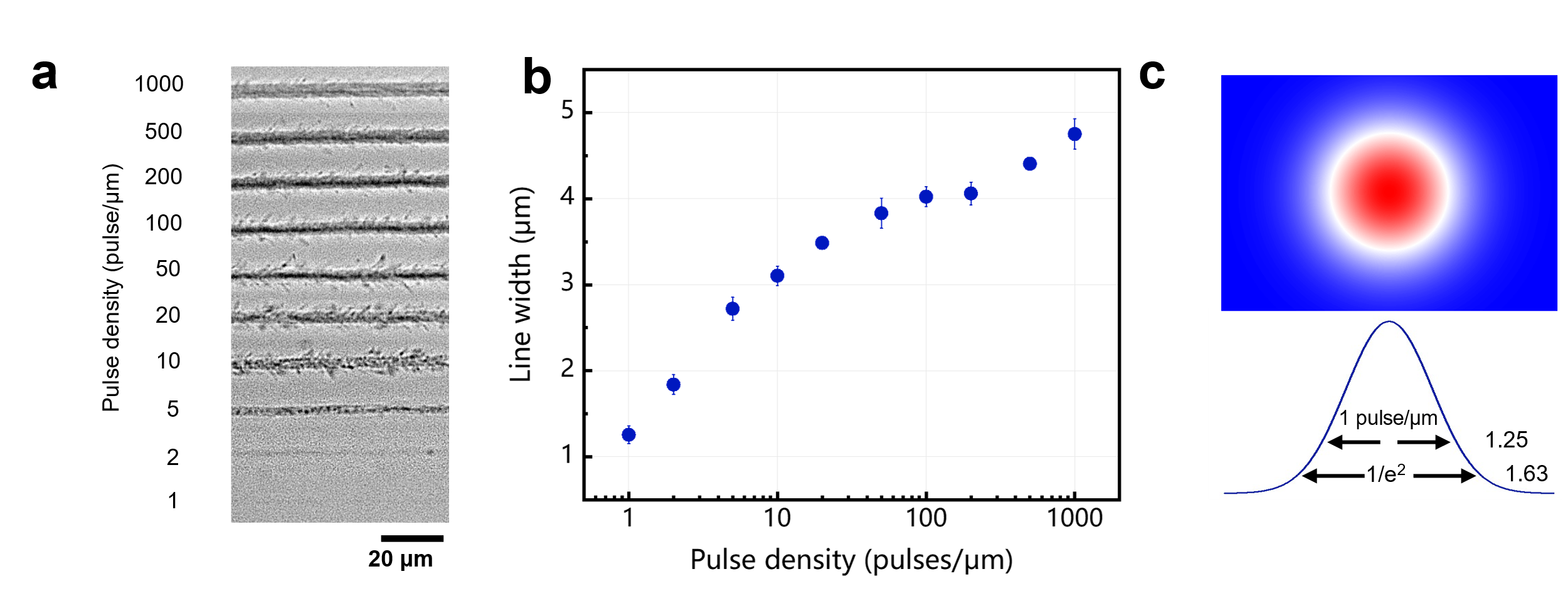
**

Fig. S1. Characterization and analysis of structures processed by single beam scanning strategy. (a) Optical microscope image of processed area by different pulse density. (b) Relationship between modified line width and pulse density. (c) Diagram of optical intensity distribution and modified region.

1. **Etching result for different etching times**

Laser scanning is done before etching process and the laser modified track is through the crystal. The etching process is performed in hot phosphoric acid with a magnetic stirrer for hours. **Fig. S2** shows the side view image of crystal dealt with wet etching. After etching for 80 hours, centimeters long channels can be seen at both side of the crystals. The average etching rate is calculated as 0.35μm·min^-1^ and 0.3 μm·min^-1^. After 200 hours etching, it can be observed that the microchannels in the crystal are almost etched to connect from both sides. The corresponding etching rate is about 0.2 μm·min^-1^. The decrease of the etch rate is mainly due to the increasing difficulty of the etching liquid into the microchannel as the depth of the microchannel increases.

**
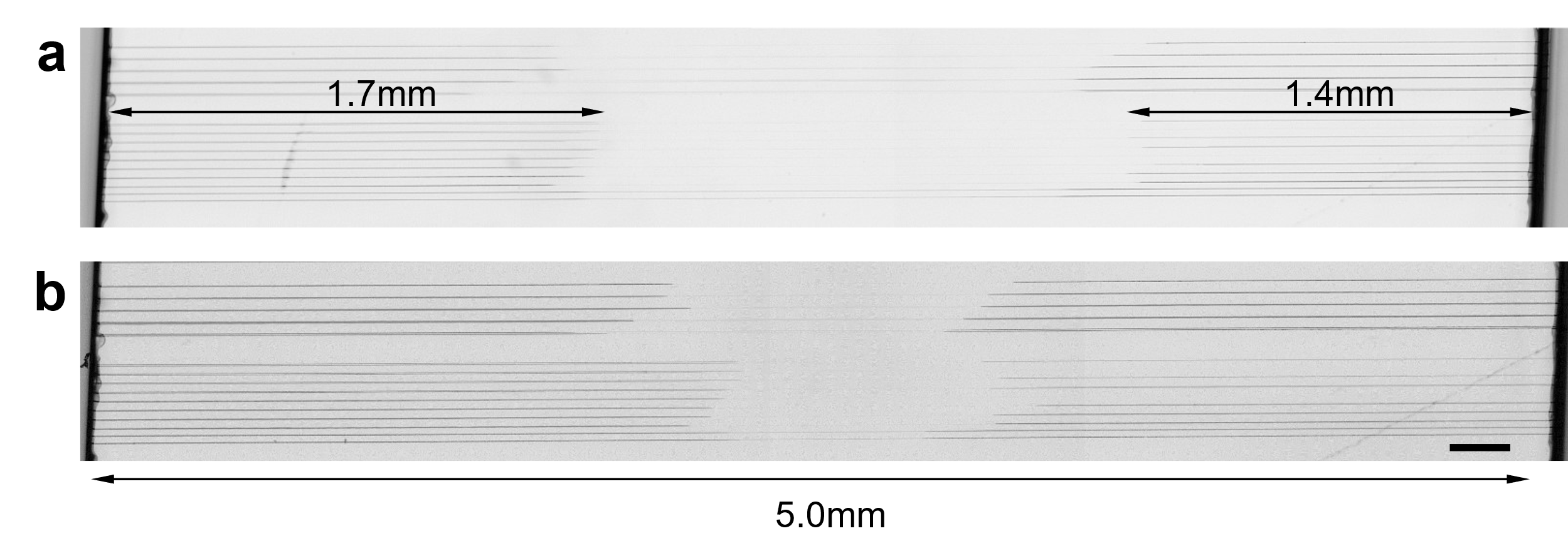
**

Fig. S2. Etching results of microchannels to centimeters length. (a) Optical microscope image of channels after etching for 80h. (b) Optical microscope image of channels after 200h. The scale bar is 200μm.

1. **Laser processed result of traditional single beam**

Traditional single focus multiple scanning strategy offers alternatives for the fabrication of photonics crystals with micro-nano gaps by changing the relative position of crystals and laser focus. There are several problems in the direct fabrication of photonics crystal elements with single focus scanning method. As can be seen in **Fig. S3a**, the first track obtained got a darker region under optical microscopy. Owing to the overlap of laser affect area, the first track suffered a secondary processing and made it different from the second one. Around the written tracks, a blue area inside the black dot line box can be observed and imply the influence of secondary scanning. It is found that the deviation of moving stage also does harm to the lattice constant control. In **Fig. S3b**, the tracks distance was varied from 1.2 μm to 1.6 μm in image i-iii respectively, but it can be seen the distance of ii is less than that of i. The deviation is mainly originated from the position error of moving stage. Besides, the straightness of laser scanning depends on the linearity of moving stage. The gap width between adjacent tracks can have fluctuations induced by the deficient linearity of device hardware.

**
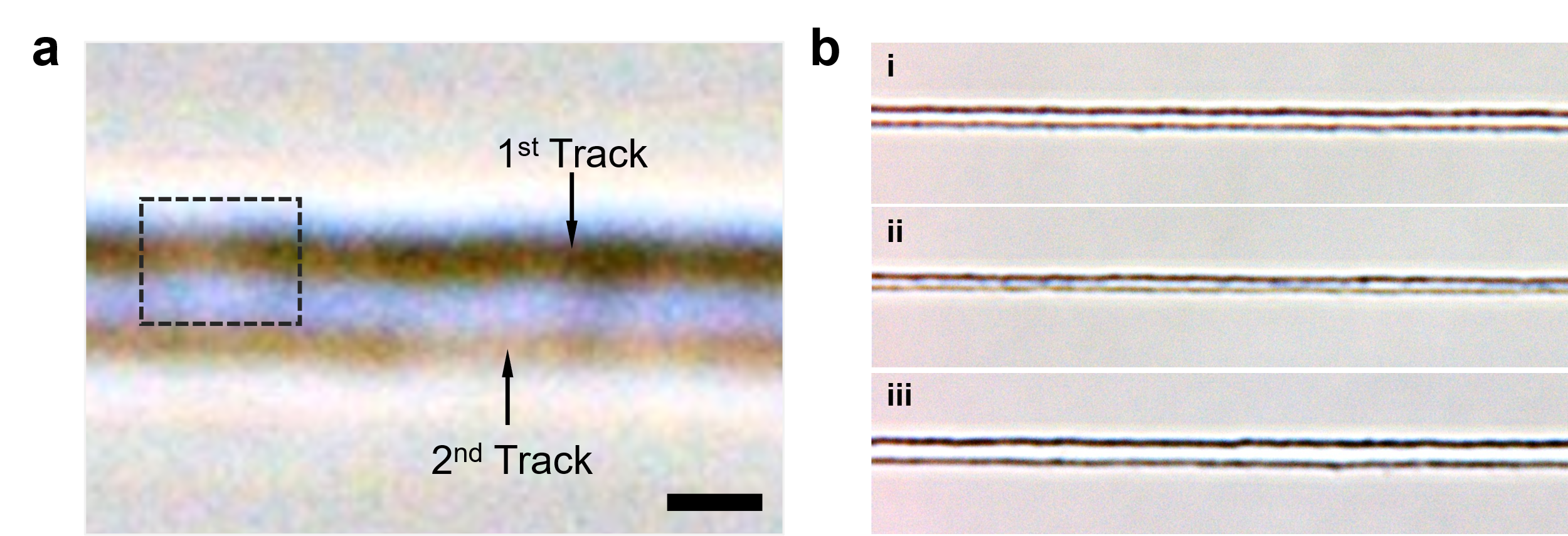
**

Fig. S3. Results before chemical etching of single beam scanning method. (a) Optical image of two channel tracks with single beam scanning method (scale bar: 1 μm). (b) Two channel tracks of different intervals (i 1.2 μm, ii 1.4 μm, iii 1.6 μm).

1. **Laser processed and etched result of multi beam laser**

The laser processed tracks and wet etching channels are shown in **Fig. S4**. With the increase of the number of phase hologram stripes, the spacing between the microchannels obtained by multi beam processing also increases. The relationship between the two is almost linear, and the theoretical and experimental results are in good agreement. From the right side of the figure, we can also observe a very interesting phenomenon. As the distance between the two laser beams increases, the z-direction length of the focal point of the laser in the x-z plane gradually decreases. Correspondingly, the width of the processed microchannel in this direction also decreases, which is consistent with the simulation results of the light field.

**
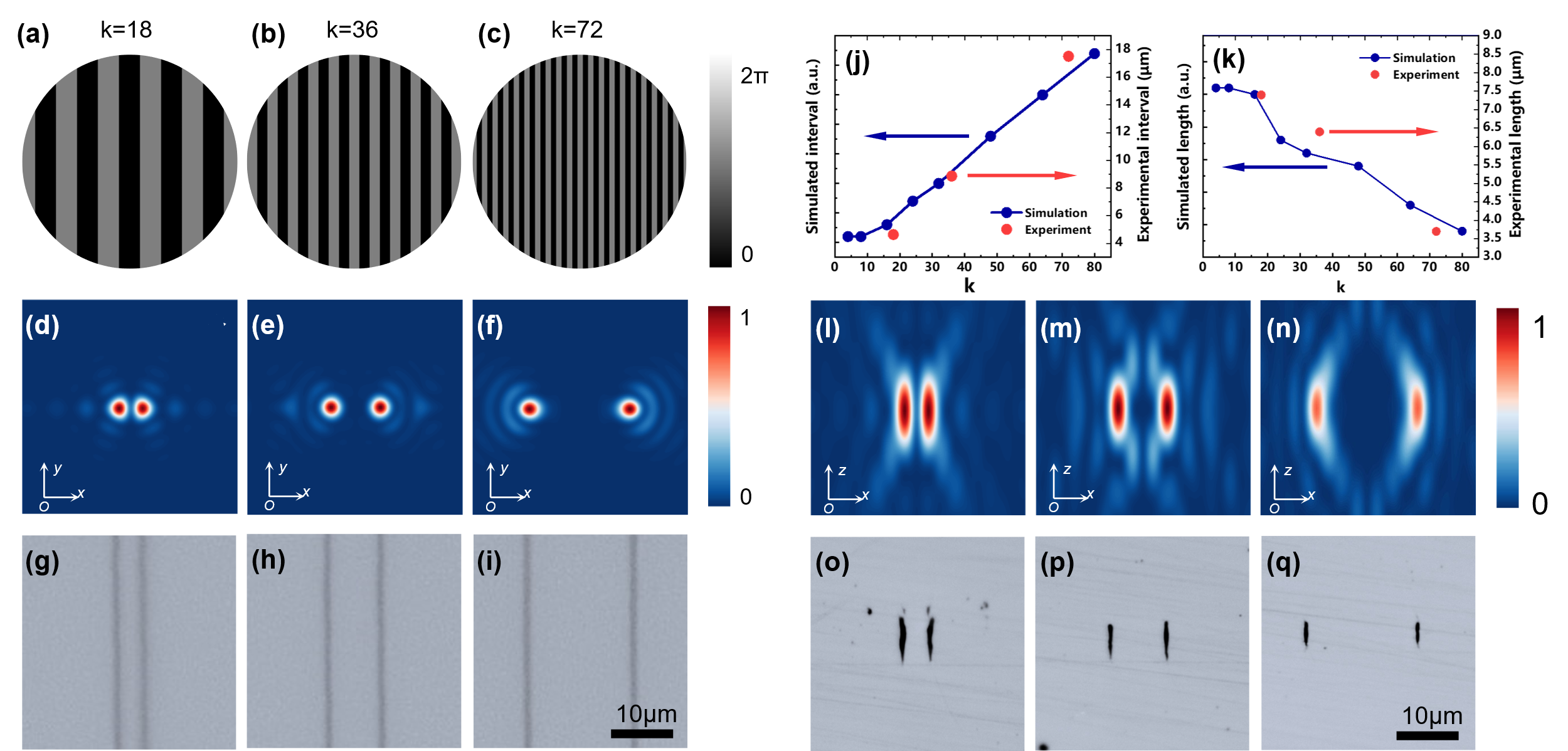
**

Fig. S4. Illustration of double-beam writing result control. (a-c) SLM holograms of different stripe numbers k for different scanning intervals. (d-f) Simulated X-Y plane optical intensity profiles of different stripe numbers k. (g-i) Optical microscope image of fabricated channels with SLM holograms in a-c. (j) Plot of the simulated and experimental intervals against the stripe numbers k. (k) Plot of the simulated and experimental lengths of the cross-section against the stripe numbers k. (l-n) Simulated X-Z plane optical intensity profiles of different stripe numbers k. (o-q) Optical microscope image of cross section of channels fabricated with SLM holograms in a-c.

1. **Control of adjacent structures with different phase grayscales**

By changing the gray value of the applied phase hologram which means changing the original integer π value to a fraction, the intensity ratio of adjacent beams can be adjusted, thereby the processing results can also be controlled. It can be seen from the **Fig. S5** that as the fractional value decreases, the intensity of one of the beams gradually decreases. Using this phase shift method, rapid processing of periodic non-uniform array structures with nano gaps can be achieved.

**
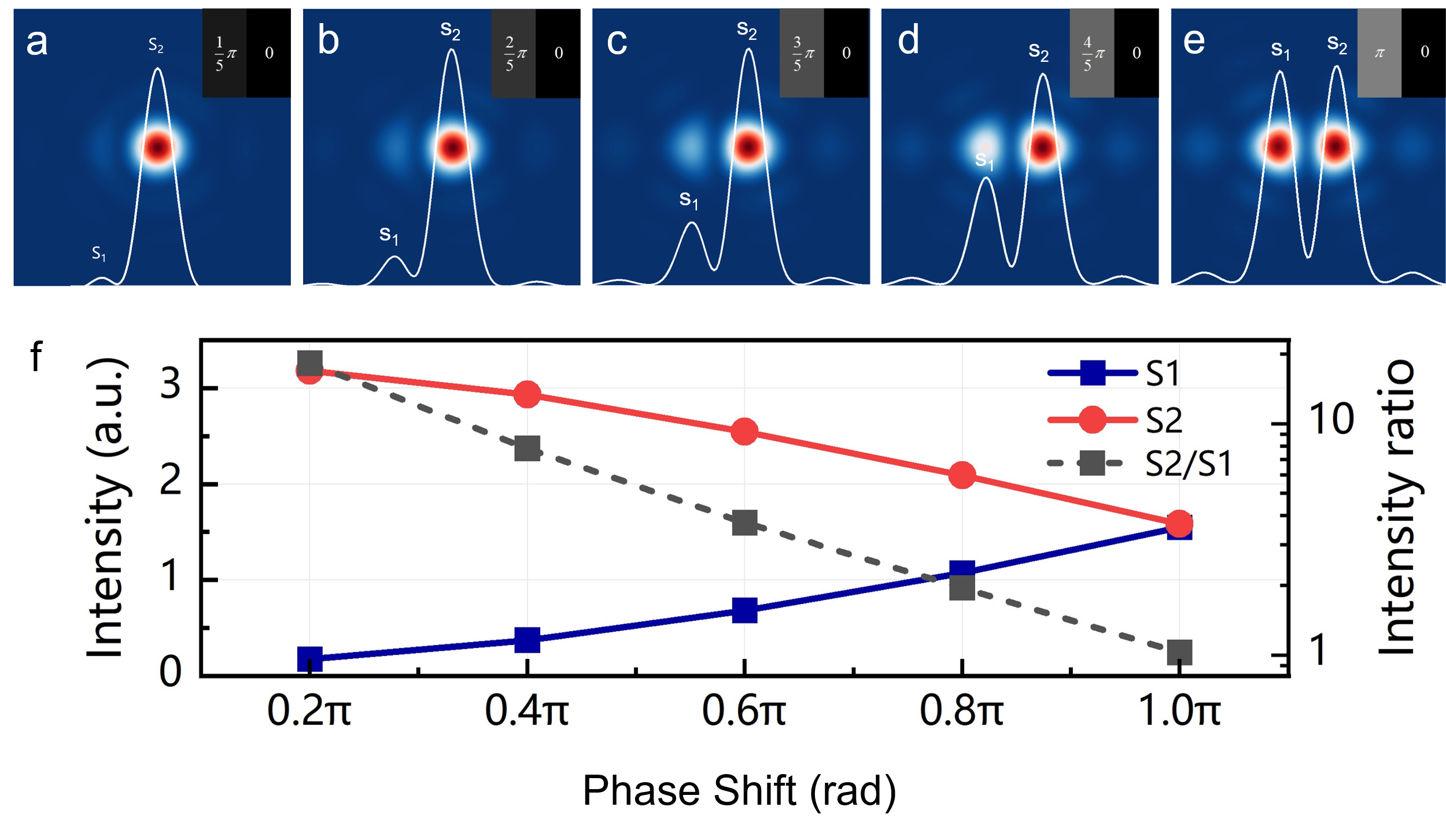
**

Fig. S5. Simulation result of optical intensity distribution with different phase shift in double beam SLM holograms. (a-e) Simulated intensity of two-foci of different phase shift. (f) Plot of intensity and intensity ratio versus phase shift.

1. **Processed results of multi beam laser writing with more focus**

**Fig. S6** shows the light field simulation and processed results for 4 and 8 beams. Phase holograms for parallel multi-beam laser writing and processed results are presented in **Fig. S6a**. The Dammann grating phase holograms were designed and corresponding light field simulation was presented based on Debye diffraction theory. Two, four and eight parallel tracks can be processed with multi-beam direct writing, respectively. After femtosecond laser inscribing, the laser modified tracks behave different refractive index compared to untreated area, which can be seen as a darker region. After phosphoric acid etching, the dark region was etched to form hollow channel which is more distinct with its surroundings as can be seen. In the side view, the cross section of etched channels also shows a uniform distribution when each channel looks almost the same from top view of optical image.

The Raman intensity spectrums of traditional single beam and proposed multi beam method are detailed presented in **Fig. S7**. There is no evident difference can be found which means that our proposed method can ensure the uniformity and stability of fabrication process and result.


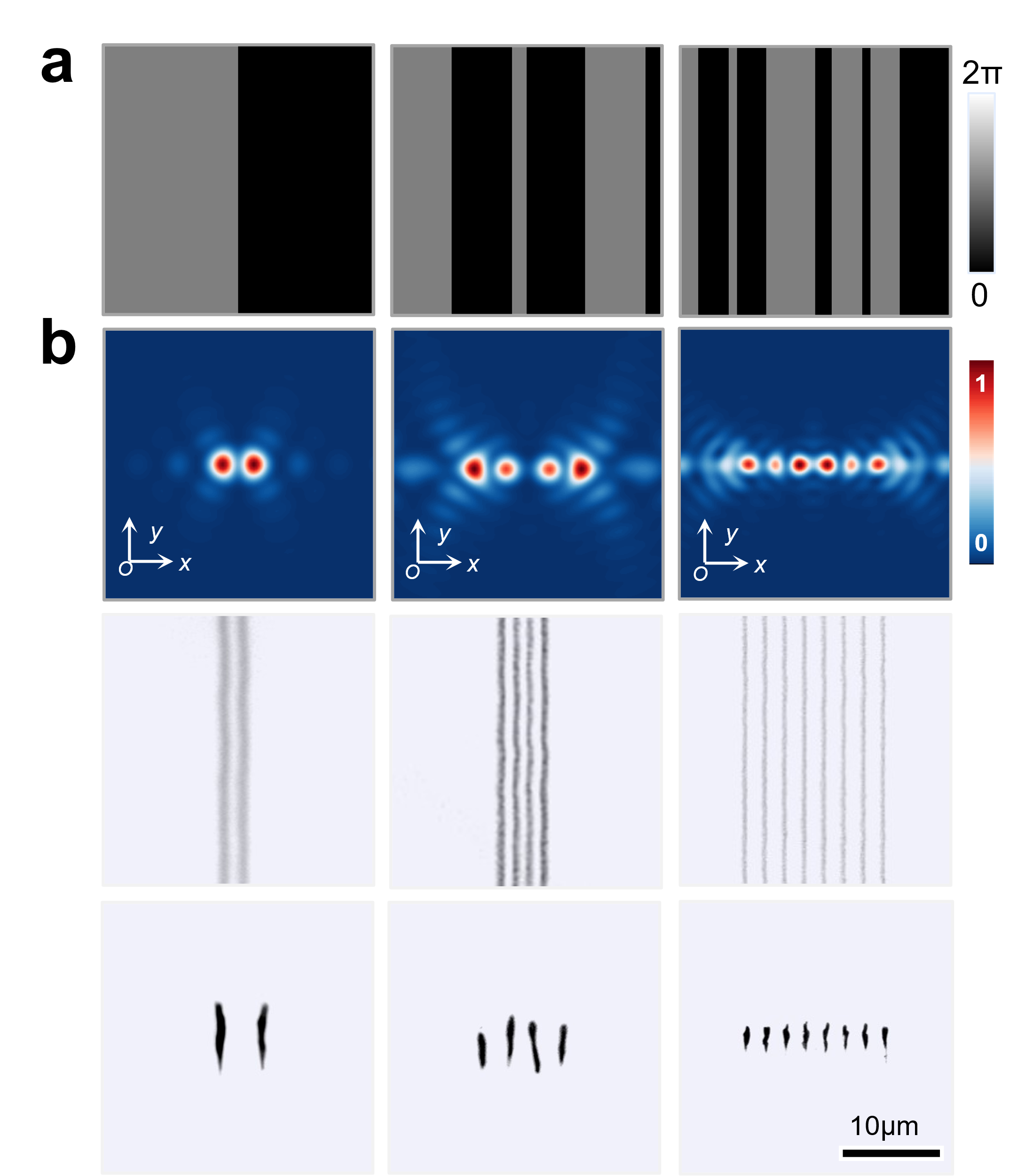


Fig. S6. Illustration of Multi-beam writing control for beam numbers L form 2-8. (a) SLM holograms of different grating phase in a single period for different beam numbers L. (b) Simulated optical intensity of different grating phases in x-y and x-z plane, optical microscope image of fabricated channels from top and side view.


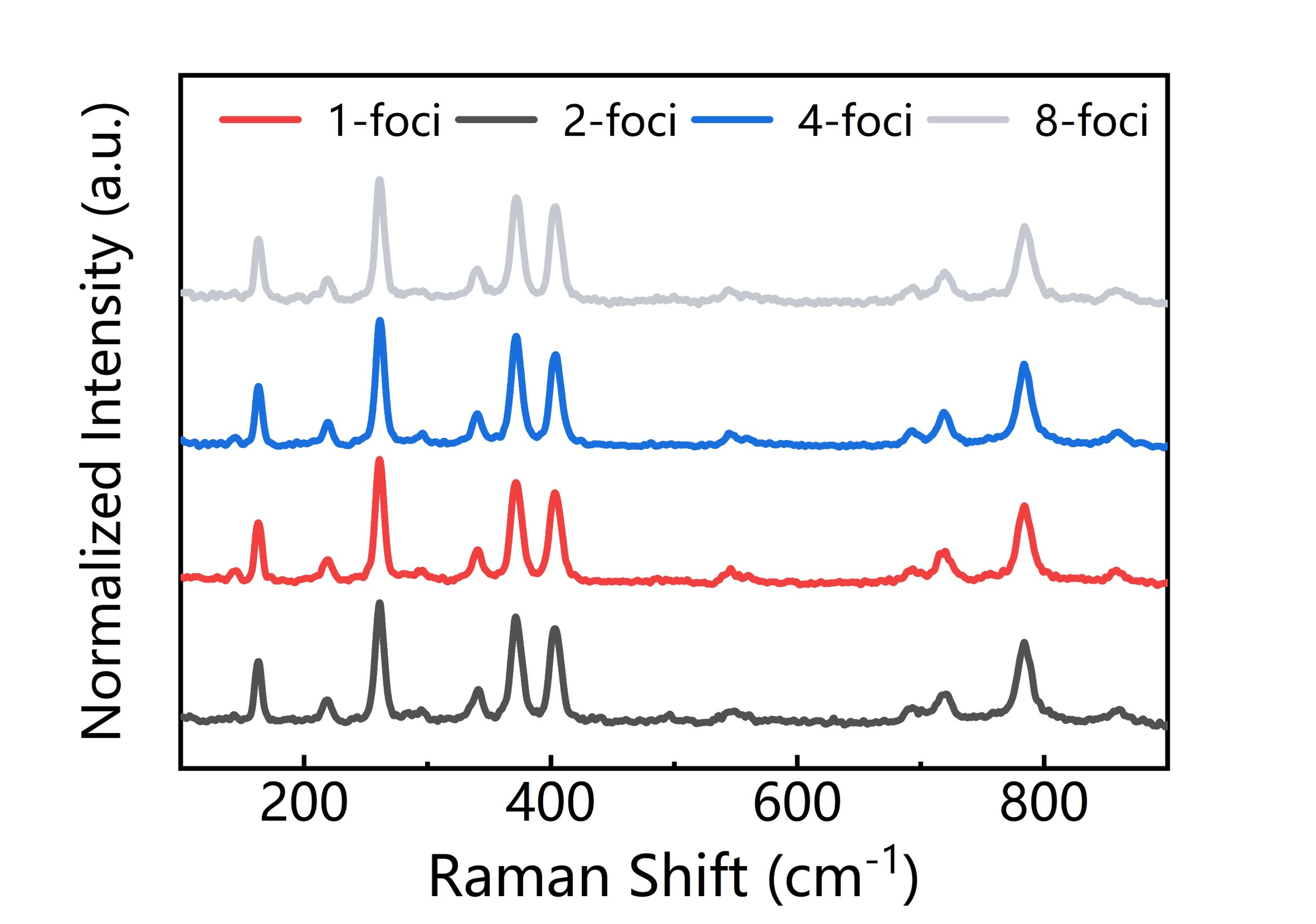


Fig. S7. Raman intensity spectrums of different beam numbers light field processed tracks in YAG crystals.

1. **Superimposing method of phase holograms**

By designing the phases applied in different SLM regions, we can obtain a new phase hologram. We call this approach SLM-split use method. There are several ways to design the division of SLM regions. Beside of stripe division, chess board like division and angle dependent division can also be used. **Fig. S8** shows the division principle of angle dependent method. The different color represents different applied phase holograms. Due to the area of sub-SLM sector is proportional to the angle, the angle of each sector needs to be specially designed to ensure that the corresponding optical intensity meet the requirements.


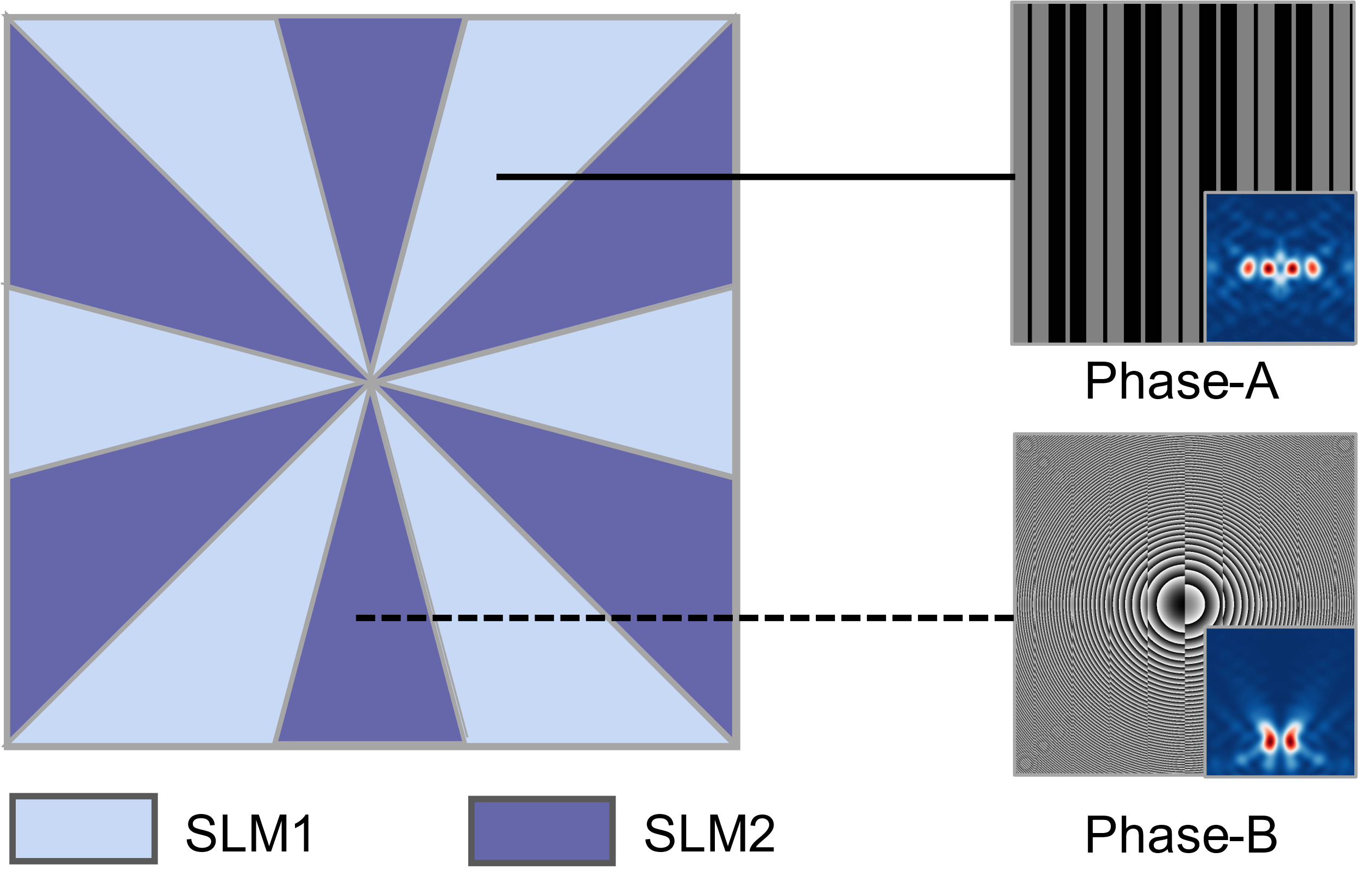


Fig. S8. Diagram for SLM-split-use strategy and holograms applied to sub-SLMs

1. **Characterization and simulation for optical gratings and waveguide structure**

**Fig. S9** displays the response of fabricated optical gratings under white light illumination. It can be observed that the crystal with grating structures shows different color when the white light incident form different angle.

A photonic crystals micro-structured optical waveguide was shown in **Fig. S10**. Hexagon distribution of hollow channels with size of 1.2×0.6 μm^2^ can be seen and the central area with a diameter of 3 μm can be used to guide incident light. The distribution section image of mode electric field component and power flow density of fabricated waveguide structure with 800 nm light luminescence was calculated and displayed.

**
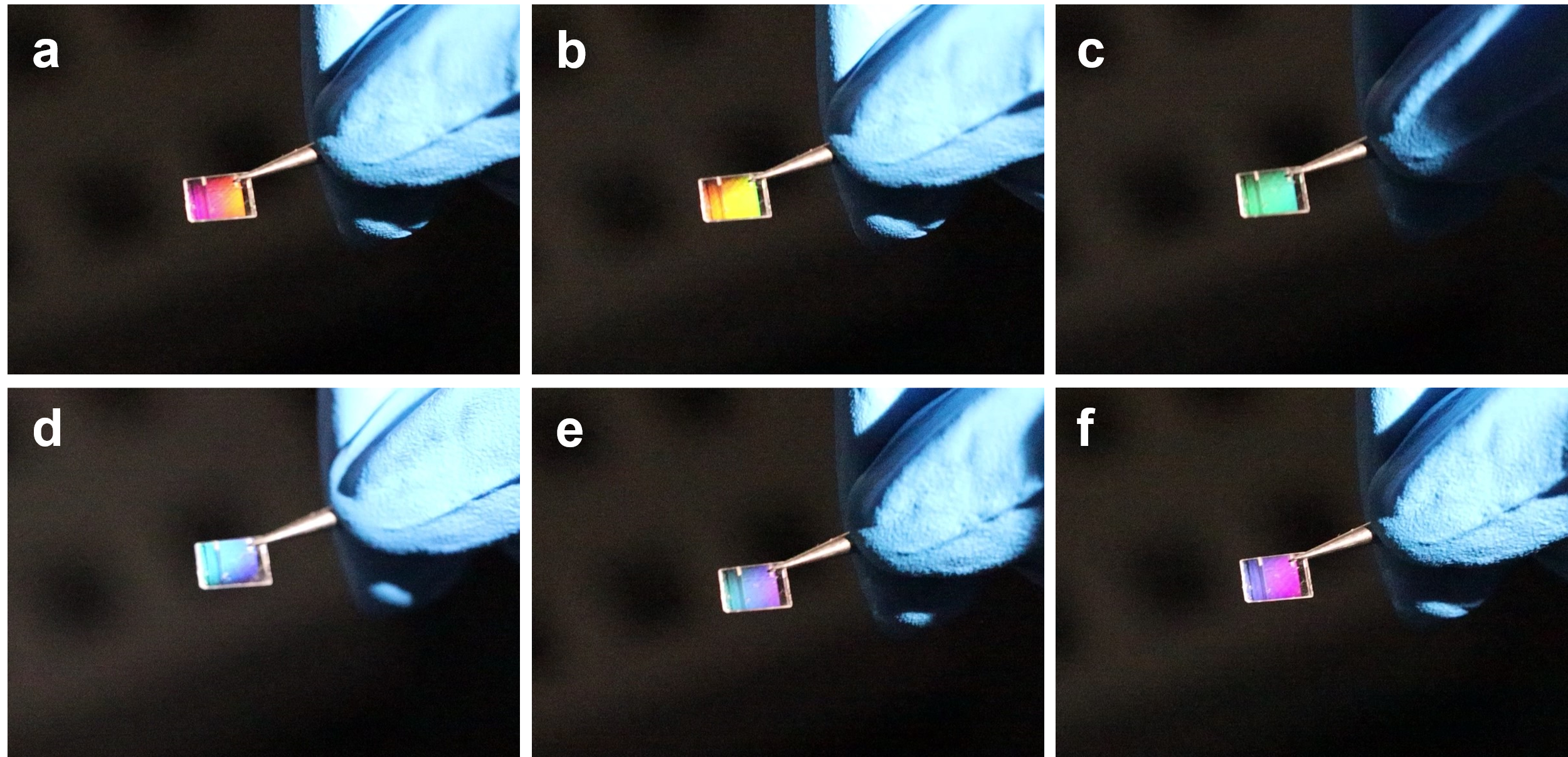
**

Fig. S9. Image of fabricated grating structure under white light illumination with different incident angles.


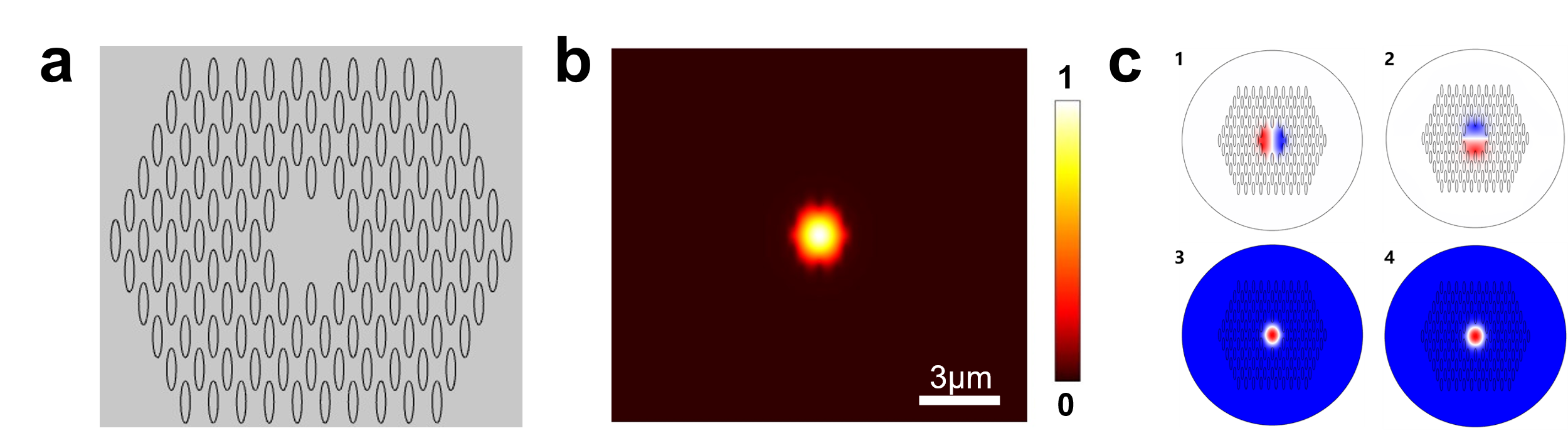


Fig. S10. (a) Schematic images of photonic crystal waveguide structure, 600nm channel interval and 200nm channel width. (b) Simulation of intensity mode profile for the waveguide structure at 800nm. (c) Distribution section image of mode electric field component and power flow density.

1. **Experimental result for faster laser scanning speed**

To balance the fabrication quality and processing speed, we conducted experiments with different laser scanning speed faster than that used in the manuscript. **Fig. S11** shows the etched channels fabricated with 200 μm·s^-1^ scanning speed. It can be found that laser can still induce crystal modification and form hollow structure after etching under faster laser processing speed.


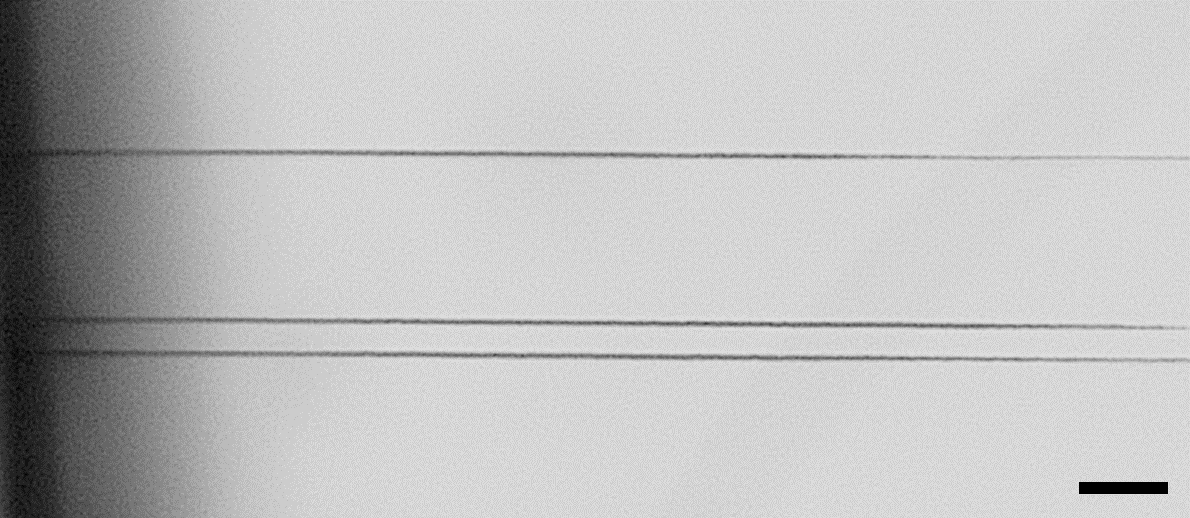


Figure S11 Optical microscope image of channels fabricated with 200 μm·s^-1^ scanning speed. The scale bar is 20 μm.

1. **SEM image of the gap between channels for accurate measurement**

To get an accurate measurement of gap between channels, we conducted a supplementary experiment and took a higher magnification and better resolution SEM image. **Fig. S12** shows the etched double channels fabricated with multi beam laser scanning method. The gap width measured is about 300 nm in the SEM image.


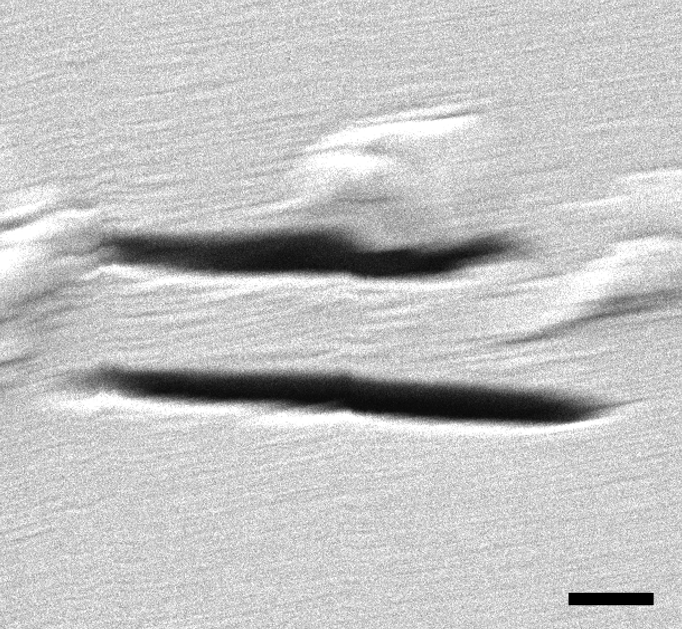


Fig. S12. Scanning electron microscope image of channels fabricated with multi beam laser scanning method. The scale bar is 250 nm.
